# Intranasal immunization with recombinant outer membrane protein A induces protective immune response against *Stenotrophomonas maltophilia* infection

**DOI:** 10.1371/journal.pone.0214596

**Published:** 2019-04-01

**Authors:** Yan Li, Xueping Tang, Zunquan Zhao, Hui Wang, Xin Wang, Xueyi Shang, Peng Liu, Zhihua Kou, Yongqiang Jiang, Yan Li

**Affiliations:** 1 No 307 Hospital of PLA of Anhui Medical University, Hefei, China; 2 Department of Critical Care Medicine, No 307 Hospital of PLA, Beijing, China; 3 Department of Respiratory and Digestive, Fengyang First People’s Hospital, Fengyang, Anhui, China; 4 The Institute of Microbiology and Epidemiology, Academy of Military Medical Sciences, Beijing, China; Universidad Nacional de la Plata, ARGENTINA

## Abstract

*Stenotrophomonas maltophilia* (*S*. *maltophilia*), a multi-drug resistant opportunistic pathogen, is associated with nosocomial and community-acquired infections. Preventive and therapeutic strategies for such infections are greatly needed. In this study, sequence alignment analysis revealed that Outer membrane protein A (OmpA) was highly conserved among *S*. *maltophilia* strains but shared no significant similarity with human and mouse proteomes. In mice, intranasal immunization with *S*. *maltophilia* recombinant OmpA (rOmpA) without additional adjuvant induced sustained mucosal and systemic rOmpA-specific antibody responses. Treatment with rOmpA stimulated significantly higher levels of secretion of IFN-γ, IL-2, and IL-17A (All *P*<0.05) from the primary splenocytes isolated from rOmpA-immunized mice than from the primary splenocytes isolated from PBS-immunized mice. Furthermore, mice immunized with rOmpA showed significantly reduced bacterial burden in the lung and reduced levels of pro-inflammatory cytokines (TNF-α and IL-6) in bronchoalveolar lavage fluid (BALF) 24 hours after intranasal *S*. *maltophilia* infection, indicating that immunization with rOmpA may have protective effects against *S*. *maltophilia* challenge in mice. Our findings suggest that intranasal immunization with rOmpA may induce mucosal and systemic immune responses in mice, trigger Th1- and Th17-mediated cellular immune responses, and thus stimulate host immune defense against *S*. *maltophilia* infection. These results also demonstrate that intranasal vaccination may offer an alternative approach to current strategies since it induces a mucosal as well as a systemic immune response.

## Introduction

*Stenotrophomonas maltophilia* (*S*. *maltophilia*), a nonfermentative Gram-negative bacillus, has been recognized as one of the emerging opportunistic pathogen in both nosocomial and community-acquired infections [1]. It has been recovered from soils and plant roots, animals, lakes, hemodialysis water and dialysate samples [1]. A significant feature of *S*. *maltophilia* is its ability to adhere to mouse tracheal mucus and thus cause respiratory tract infections [2]. *S*. *maltophilia*-associated infection often causes high mortality, especially in immunocompromised patients [3]. In addition to the most common clinical presentation pneumonia, *S*. *maltophilia* infection can also cause bacteremia, urinary tract infections, meningitis, septic arthritis, endocarditis, and soft-tissue infection [4–10]. *S*. *maltophilia* resists antibiotics by intrinsic and acquired resistance mechanisms, such as poor membrane permeability to antibiotics and overexpression of multidrug resistance efflux pumps, β-lactamases, and antibiotics-modifying enzymes [11]. Thus, compared to antibiotic treatment, vaccination appears to be a more promising method to control *S*. *maltophilia* infection. However, effective vaccine against *S*. *maltophilia* infection is still unavailable.

Outer membrane proteins (Omps) are major surface proteins and antigens of Gram-negative bacteria and contribute substantially to induce host immune response [12–16]. In channel catfish, Omps are involved in inducing host defense against *S*. *maltophilia* infection [17]. Thus, Omps could be promising vaccine candidates. Previous studies have shown that compared with vaccines prepared from Omp complexes, Omp subunit vaccines are safer, more effective, and more easily for mass production [16, 18]. In our preliminary study, we used immunoproteomic approach to discover that outer membrane protein A (OmpA) was a strong immunogenic antigen and could be a good vaccine candidate against *S*. *maltophilia* infection [19]. OmpA and OmpA-like proteins, such as OmpA in *Escherichia coli* (*E*. *coli)* [20], OmpA in *Klebsiella pneumonia* [21], OmpA in *Acinetobacter baumannii* [22], and OprF in *Pseudomonas aeruginosa* [23] have been tested as vaccines against bacterial infections. MopB, a member of the OmpA family, is expressed the most abundantly on the cell surface of *S*. *maltophilia* and contribute to maintenance of cell membrane structure, and bacterial adhesion, and *S*. *maltophilia* with the MopB gene deletion cause milder L929 cell cytotoxicity compared with the wild type bacteria [24]. However, whether OmpA in *S*. *maltophilia* could be an effective vaccine against *S*. *maltophilia* infection remains unclear.

An ideal vaccine should be able to elicit sustained potent host defense locally at the infection site and systemically [25]. The initial infection route of *S*. *maltophilia* is usually at mucosal sites [2]. Thus, vaccines that can induce strong systemic immune responses but fail to stimulate host immune responses at mucosal sites may not be effective against *S*. *maltophilia* infection. Compared with other injection routes, such as subcutaneous, intramuscular and peritoneal injection, the intranasal route can induce both mucosal and systemic immune responses effectively [26]. Additionally, intranasal route has other advantages, such as an ease access to the respiratory tract and low onset-dose of an antigen [27]. Therefore, intranasal delivery is likely to improve the effectiveness of immunization against *S*. *maltophilia* infection. Previous studies have found that intranasal immunization with OmpA of *Shigella flexneri* 2a can induce protective immune response in a mouse model [28], and mucosal immunization with OmpA can stimulate host defense against multidrug-resistant *Acinetobacter baumannii* infection [29]. Therefore, an intranasal delivery of OmpA as a vaccine could elicit effective systemic and mucosal immune responses against *S*. *maltophilia* infection. OmpA appears as a new type of pathogen-associated molecular pattern (PAMP) usable as a vector in anti-infectious [30]. Therefore, we investigate the protective efficacy of intranasal immunization with rOmpA without additional adjuvant against *S*. *maltophilia*.

In this study, we cloned the *ompA* gene of *S*. *maltophilia* strain K279a and expressed and purified recombinant OmpA (rOmpA) protein successfully. The rOmpA-induced hose defense against *S*. *maltophilia* infection and the underlying mechanism were investigated in a mouse model and primary mouse splenocytes.

## Materials and methods

### Bacterial strains and mice

*S*. *maltophilia* strain K279a was purchased from the American Type Culture Collection (ATCC) and routinely grown in lysogeny broth (LB) or on LB agar plates at 37°C. Because the whole genome of *S*. *maltophilia* K279a has been sequenced and *S*. *maltophilia* K279a was considered as a representative genome sequence strain [31], we used this strain for the study. *E*. *coli* BL21 (DE3) (Novagen) was used for expressing recombinant protein. Specific pathogen-free female C57BL/6 mice were housed at the animal care center of the Academy of Military Medical Sciences (AMMS), China. All mice were matched for age and maintained in a specific-pathogen free facility before being challenged by bacterial infection. All experiments were handled according to protocols approved by Institutional Animal Care and Use Committee of AMMS.

### Bioinformatic analysis of the OmpA protein

The amino acid sequence of OmpA from *S*. *maltophilia* K279a strain was used as a reference for the alignment analysis [Basic Local Alignment Search Tool (BLAST) analysis] as described previously [22]. Amino acid sequences of OmpA were manually aligned using ALIGNX (Vector NTI package version 9.0). Antigenicity was analyzed as described [32]. Antigen probability of OmpA was estimated by VaxiJen at http://www.ddg-pharmfac.net/vaxijen/VaxiJen/VaxiJen.html. Hydrophilicity, flexibility, and surface accessibility of OmpA were predicted by using the IEDB tools at http://tools.iedb.org/bcell/.

### Preparation of recombinant OmpA (rOmpA)

The strategy for the construction, expression, and purification of the rOmpA was similar to the previous description [29]. Briefly, the *ompA* gene of *S*. *maltophilia* strain K279a was amplified and cloned into the expression vector pET-30a (+) (Takara, Japan). BL21 (DE3) cells transformed with the recombinant vector were grown in LB medium supplemented with kanamycin (50μg/mL) at 37°C until an optical density (OD_600_nm) of 0.4–0.6 was achieved. rOmpA expression was induced by 1 mM isopropyl-β-D-thiogalactopyranoside (IPTG) (Sigma) at 30°C for 6h. The bacterial pellets were collected and lysed by sonication on ice. After centrifugation at 8000rpm 4°C for 15min, the supernatant was harvested. Subsequently, the 6×-His tagged recombinant protein was purified with a HiTrap Chelating HP column (GE Healthcare) according to the manufacturer’s instructions. Lipopolysaccharide (LPS) contamination was then removed from the recombinant protein preparation using the Toxin Eraser endotoxin removal kit (Genscript Biotechnology, Inc.), and the residual LPS level was determined using the Chromogenic End-point Endotoxin Assay Kit (Chinese Horseshoe Crab Reagent Manufactory, Xiamen, China). Purified rOmpA was stored at -80°C before use.

The expression and purity of rOmpA were analyzed by 12% sodium dodecyl sulfate-polyacrylamide gel electrophoresis (SDS-PAGE) and Coomassie blue staining. After electrophoresis, rOmpA was also transferred to polyvinylidene difluoride (PVDF) membranes, followed by blocking in 3% bovine serum albumin (BSA) for 2 hours at room temperature. After washing, the membrane was incubated with mouse pre-immune serum (1:2000), mouse anti-inactivated *S*. *maltophilia* serum (1:2000), or mouse anti-His primary antibody (1:2000) at 4°C overnight. Then, the membranes were incubated with a goat IRDye680RD-labelled anti-mouse IgG antibody (1:10000) (LI-COR) for 1 hour at room temperature. The blot was imaged using the LI-COR Odyssey Infra-Red Imager.

### Immunization protocols and sample collection

In this study, 5-6-week-old female C57BL/6 mice with similar body weight were randomized into rOmpA group and control group. Typically, the mice in rOmpA group were intranasally immunized three times at 7-day intervals with 25μL sterile phosphate buffered solution (PBS) (Gibco) containing 20μg rOmpA. The control mice were immunized with PBS instead. Blood and bronchoalveolar lavage fluid (BALF) were collected from mice at 7, 14, 21, 50, and 80 days after the first intranasal immunization. The BALF was obtained by lavaging the lung three times with 1mL sterile PBS. The serum samples were centrifuged at 4,000 × g for 10 min, and the BALF supernatants were centrifuged at 2,000 × g for 10 min. The supernatants were harvested. Oral, intestinal, and urethral washes were obtained from mice at 7 days after the last intranasal immunization according to a previously described method [33]. Oral, intestinal, and urethral washes were centrifuged at 2,000 × g for 10 min and the supernatants were harvested. All of these samples were collected in Eppendorf tubes and stored at -80°C for further studies.

### Detection of anti-rOmpA antibodies by ELISA

The levels of anti-rOmpA antibodies in serum, BALF, and other mucosal washes samples were determined by enzyme-linked immunosorbent assay (ELISA). Briefly, 96-well plates were coated with rOmpA at a concentration of 0.5μg/mL overnight at 4°C and blocked with 3% BSA in PBS for 2 h at 37°C. The serum samples were added to wells at a dilution of 1:10000, while oral, intestinal, urethral washes and BALF samples were added to wells at a dilution of 1:2. HRP-conjugated goat anti-mouse IgG, IgG_1_, IG_2a_ (diluted 1:8000) (Santa Cruz) and goat anti-mouse IgA (diluted 1:8000) (Santa Cruz) were used as the secondary antibody. Color was developed by adding 100μL 3, 3’, 5, 5’- tetramethylbenzidine (TMB) (Solarbio, China). The plates were kept at 37°C for 15 min and the color development was stopped by adding 100μL 1mmol/ mL H_2_SO_4_ in each well. The absorbance of the reaction system was measured at 450 nm by using a microplate reader (Thermo Fisher).

### Analysis of cellular immune response

In order to evaluate the cellular immune response induced by rOmpA, we isolated primary splenocytes. Mouse spleens isolated from mice 7 days after the last immunization were gently ground, and the spleen tissue homogenate were then filtered through a stainless steel mesh to collect splenocytes. The splenocytes were then washed, re-suspended, and seeded in 24-well culture plates at a density of 3 × 10^6^ cells per well. The cells were cultured in RPMI 1640 (Gibco) supplemented with 10% fetal bovine serum and 100U/mL streptomycin (Sigma), 100U/mL penicillin (Sigma) at 37°C in a 5% CO_2_ incubator. After 4 h of incubation, the cells were stimulated with 5μg rOmpA. The levels of gamma interferon (IFN-γ), interleukin-2 (IL-2), interleukin-4 (IL-4), and interleukin-17A (IL-17A) in the harvested cell-free supernatants at 12, 24, 48, and 72 h after adding rOmpA were detected using ELISA kits (Neobioscience, China) according to the manufacturer’s instructions.

### *S*. *maltophilia* infection challenge in mice

As mentioned above, two groups of female C57BL/6 mice were immunized with PBS or rOmpA. Ten days after the final immunization, 10 mice from each group were challenged with intranasal administration of 25μL *S*. *maltophilia* K279a suspension at a dose of 4×10^9^ CFU/mL. Twenty-four hours after the challenge, mice were sacrificed and the lungs were removed aseptically and homogenized in sterile PBS immediately. Samples were serially diluted with sterile PBS and plated on LB agar medium. After overnight incubation, the colonies on agar plates were counted and the bacterial loads in lung were calculated. For pro-inflammatory cytokine assay, mice were sacrificed and BALF was collected at 6 h and 24 h post-challenge. The levels of TNF-a and IL-6 in BALF were measured by sandwich ELISA kits (Neobioscience, China) according to the manufacturer’s instructions.

## Statistical analysis

Comparison between the rOmpA group and control group was analyzed by two-tailed Student’s t-test or Mann-Whitney rank test (for bacterial loads and levels of pro-inflammatory cytokines after the bacterial challenge) using GraphPad Software Prism 5. Data are presented as mean ± standard deviation (SD) or median with interquartile range where applicable. The presented results were from at least three independent experiments. Statistical significance was assumed at p<0.05.

## Results

### Sequence analysis of OmpA

The amino acid sequence of OmpA from *S*. *maltophilia* strain K279a was obtained from NCBI. The OmpA protein of *S*. *maltophilia* K279a, deduced from the gene (1,098 bp; Smlt0955), is a 366-amino acid protein with an N-terminal signal peptide of 22 amino acids and a conserved C-terminal OmpA domain. BLAST analysis revealed that OmpA shared 86% to 99% sequence identity among *S*. *maltophilia* strains and no significant similarity with human and mouse proteomes. Besides, OmpA is widely present in *Acinetobacter baumannii*, *Pseudomonas aeruginosa*, *E*. *coli*, and *Klebsiella pneumoniae* strains. Sequence alignment revealed that *S*. *maltophilia* K279a OmpA shared a low degree of identity with OmpA from *Acinetobacter baumannii* (19.8%; AAR83911.1), OprF from *P*. *aeruginosa PA01* (31.6%; NP_250468.1), OmpA from *E*. *coli* (21.7%; WP_011703519.1), and OmpA from *Klebsiella pneumonia* (21.3%;). Antigen probability of OmpA was predicted as 0.7119. The average score of flexibility, hydrophilicity, and surface accessibility for the designed construct were 1.005, 2.238, and 1.000, respectively.

### Expression and purification of rOmpA

**Fig 1:**
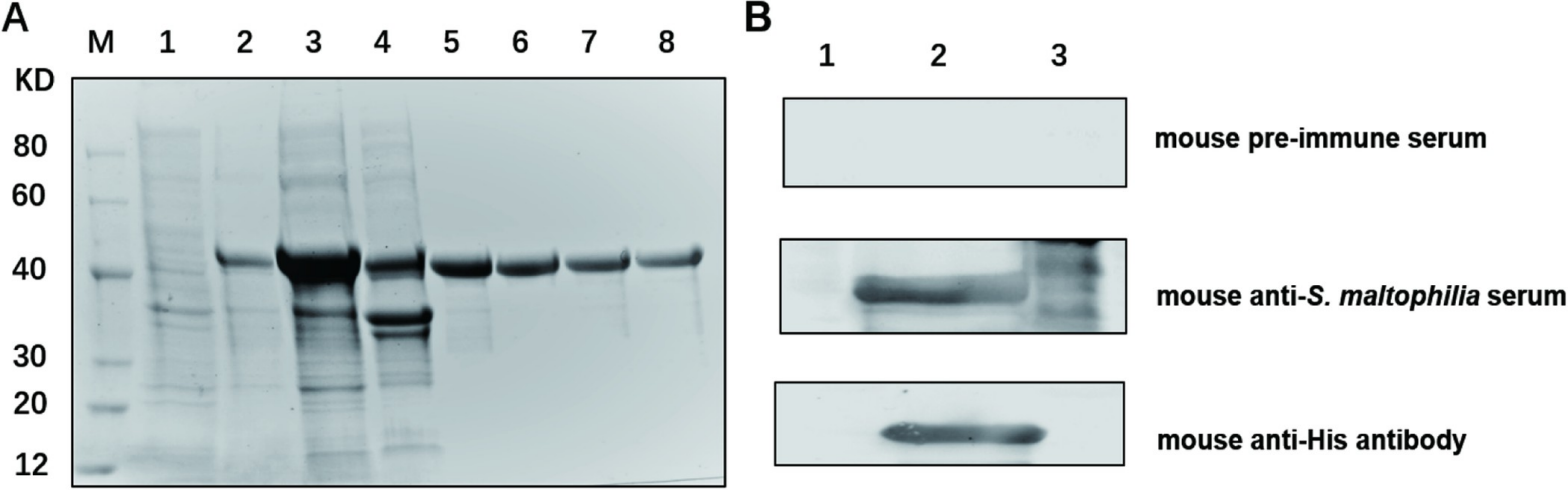
Representative images of SDS-PAGE and western blotting analysis of OmpA protein. **A:** Recombinant OmpA protein was expressed in BL21 and purified using nickel-chelating chromatography. The eluted proteins were subjected to SDS-PAGE and detected by direct staining with Coomassie brilliant blue. Lane M, protein marker (12-80kDa); lane 1, total proteins of uninduced BL21 harboring pET-30a(+)-ompA; lane 2, total proteins of induced BL21 harboring pET-30a(+)-ompA; lane 3, supernatants of induced BL21 harboring pET-30a(+)-ompA; lane 4, precipitation of induced BL21 harboring pET-30a(+)-ompA; lanes 5–8, purified rOmpA (40 kDa). **B:** Western blotting analysis of rOmpA using mouse pre-immune serum, mouse anti-inactivated *S*. *maltophilia* serum, and mouse anti-His antibody. Lane 1, total proteins of induced BL21 harboring pET-30a(+); lane 2, rOmpA; lane 3, total proteins of *S*. *maltophilia* strain K279a.

rOmpA was expressed in *E*. *coli* BL21 (DE3) system and purified through a nickel-chelating chromatography successfully. The SDS-PAGE showed that the purified protein was approximately 40 kDa, which is consistent with the calculated molecular weight of rOmpA protein with a poly-histidine tag (**Fig 1A**). The purity of rOmpA was above 90%, as determined by SDS-PAGE with Coomassie blue staining. Immunoblot analysis demonstrated that the purified rOmpA protein reacted with mouse anti-inactivated *S*. *maltophilia* serum and mouse anti-His antibody (**Fig 1B**). The total proteins of *S*. *maltophilia* strain K279a reacted with mouse anti-inactivated *S*. *maltophilia* serum, whereas the total proteins of *E*.*coil* harboring the empty plasmid induced by IPTG did not show any significant reaction (**Fig 1B**).

### Mouse mucosal immune response induced by intranasal immunization with rOmpA

**Fig 2:**
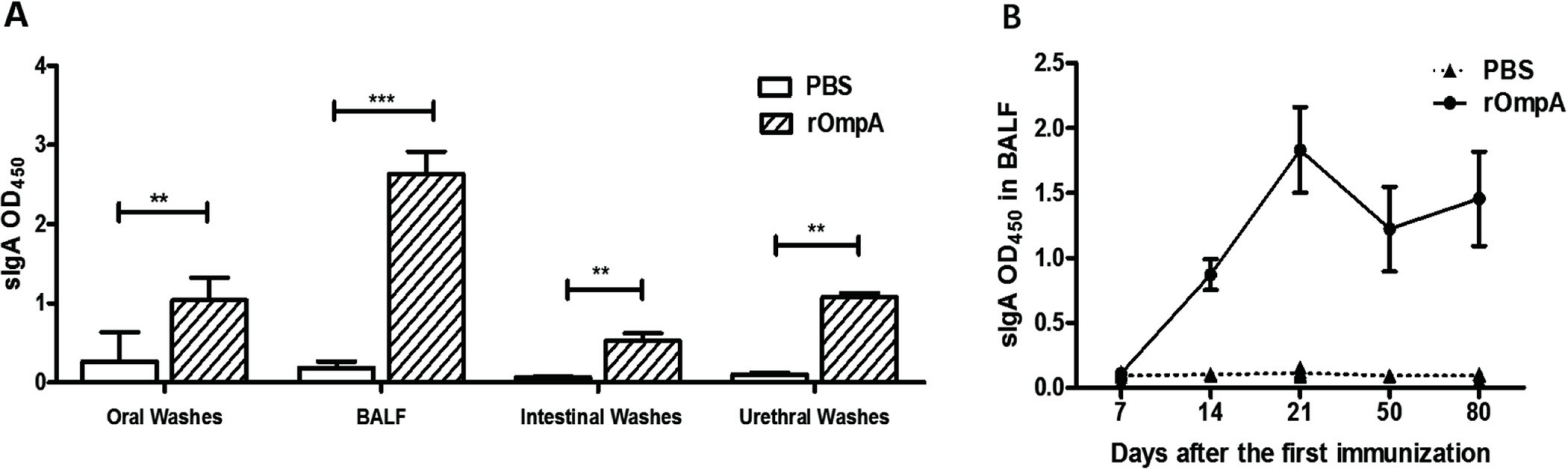
Mucosal sIgA antibody levels in the mucosal washes. **A:** sIgA antibody levels in the oral, intestinal, urethral washes, and BALF from rOmpA-immunized group and control group (n = 5) were measured at 7 days after the last immunization. **B:** sIgA antibody levels in BALF were detected at 7, 14, 21, 50, and 80 days after the first immunization from mice (n = 5) intranasally immunized with rOmpA or PBS. Data are expressed as mean ± SD. Results are representative for three independent experiments. ** *P* < 0.01, *** *P* < 0.001 for the rOmpA group compared with the control group was analyzed by Student’s t-test (two-tailed).

We determined the levels of secretory IgA (sIgA) in oral, intestinal, urethral washes and BALF to estimate mucosal immune response to intranasal administration of rOmpA. Seven days after the last immunization, the levels of sIgA in oral, intestinal, and urethral washes and BALF from the mice immunized with rOmpA were significantly higher than those from the control mice (P<0.01; **Fig 2A**). Besides, the immune response, which was represented by the increased sIgA levels, remained at high levels till 80 days after the first immunization (**Fig 2B**). The sIgA levels in BALF from the rOmpA group increased continuously during immunization and reached a peak at 21 days after the first immunization (**Fig 2B**). In contrast, rOmpA-induced mucosal sIgA antibody was not detected in BALF from mice immunized with PBS (**Fig 2B**).

### Mouse systemic humoral immune response induced by intranasal immunization with rOmpA

**Fig 3:**
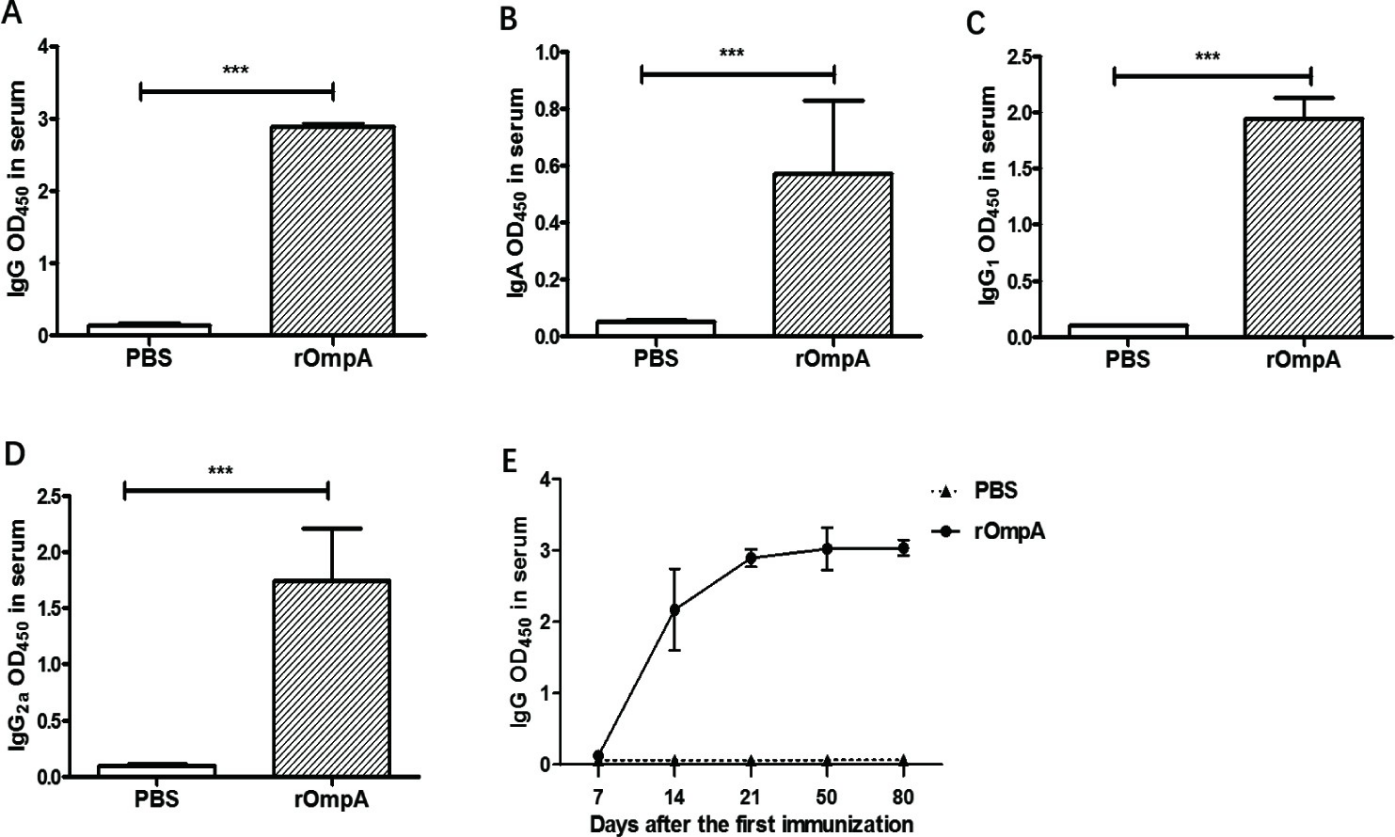
Systemic antibody levels in serum from mice intranasally immunized with rOmpA or PBS. IgG (**A**) and IgA (**B**) antibody levels in serum were detected 7 days after the last immunization from the rOmpA mice and control mice (n = 10). IgG_1_ (**C**) and IgG_2a_ (**D**) antibody levels in serum were also detected 7 days after the last immunization from the rOmpA-immunized mice and control mice (n = 9) (**C** and **D**). **E**. IgG antibody levels in serum were detected at 7, 14, 21, 50, and 80 days after the first immunization from the rOmpA-immunized mice and control mice (n = 5). Data are shown as mean ± SD. The results are representative for at least three independent experiments. *** *P* < 0.001 for the rOmpA group compared with control group was analyzed by Student’s t-test (two-tailed).

In order to assess the humoral immune response in mice, we measure rOmpA-induced IgG and sIgA levels in serum samples. Compared with mice immunized with PBS, mice with intranasal immunization of rOmpA showed significantly increased serum levels of IgG (**Fig 3A**), IgA (**Fig 3B**), IgG_1_ (**Fig 3C**), and IgG_2a_ (**Fig 3D**) at 7 days after the last immunization (All *P*<0.001). Besides, serum IgG remained at high levels in mice with intranasal immunization with rOmpA even two months after the last immunization (**Fig 3E**).

### Cellular immune response induced by intranasal immunization with rOmpA

**Fig 4:**
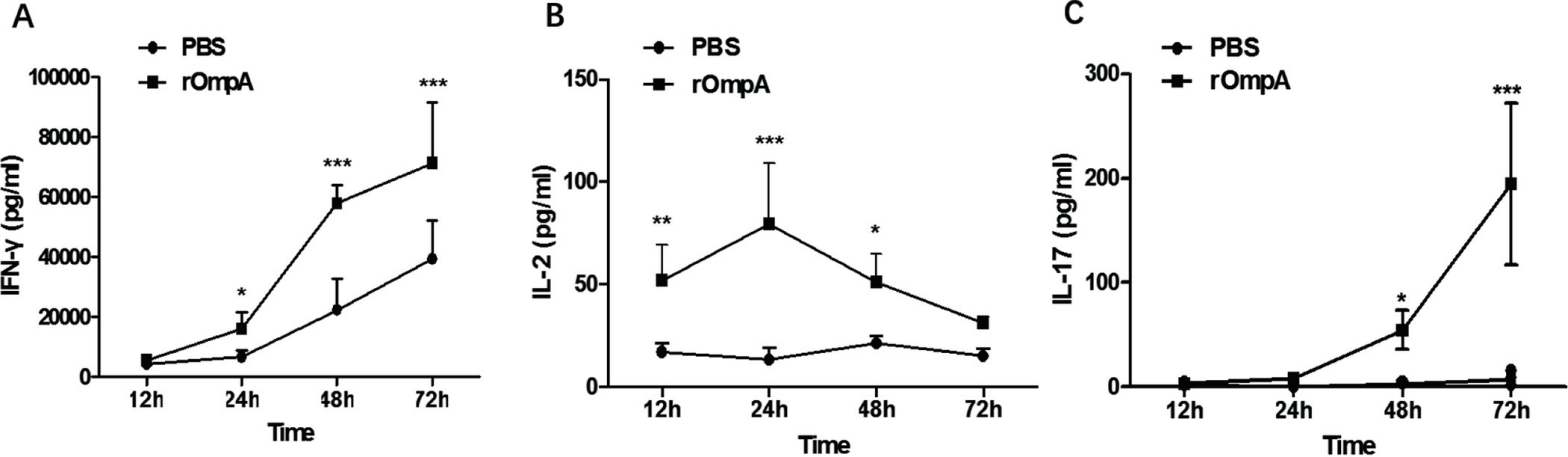
Effect of intranasal immunization with rOmpA on cytokine production from primary mouse splenocytes. Primary splenocytes were harvested from mice immunized with rOmpA or PBS for the detection of IFN-γ (**A**), IL-2 (**B**), and IL-17A (**C**) levels by ELISA. Data are shown as the mean ± SD (n = 4). Results are representative for three independent experiments. * *P* <0.05, ** *P* < 0.01, *** *P* < 0.001 for the rOmpA group compared with control group was analyzed by Student’s *t*-test (two-tailed).

We subsequently evaluated the cellular immune response to rOmpA by analyzing cytokine production of primary splenocytes isolated from mice 7 days after the first immunization. The primary splenocytes were isolated from control and rOmpA-immunized mice and then stimulated *in vitro* with rOmpA (5μg/mL). The levels of IFN-γ, IL-2, IL-4, and IL-17A in the culture media were analyzed by ELISA. The levels of IFN-γ (**Fig 4A**) and IL-2 (**Fig 4B**) in the culture supernatant from the rOmpA group were significantly higher than those from the control group (All *P*<0.05), whereas the IL-4 levels were lower than the limit of detection in this experiment. Besides, 48 and 72 hours after the treatment with rOmpA, the primary splenocytes isolated from rOmpA-immunized mice secreted significantly higher levels of IL-17A than the primary splenocytes isolated from control mice (All *P*<0.05, **Fig 4C**).

### rOmpA immunization protected mice against respiratory *S*. *maltophilia* challenge

**Fig 5:**
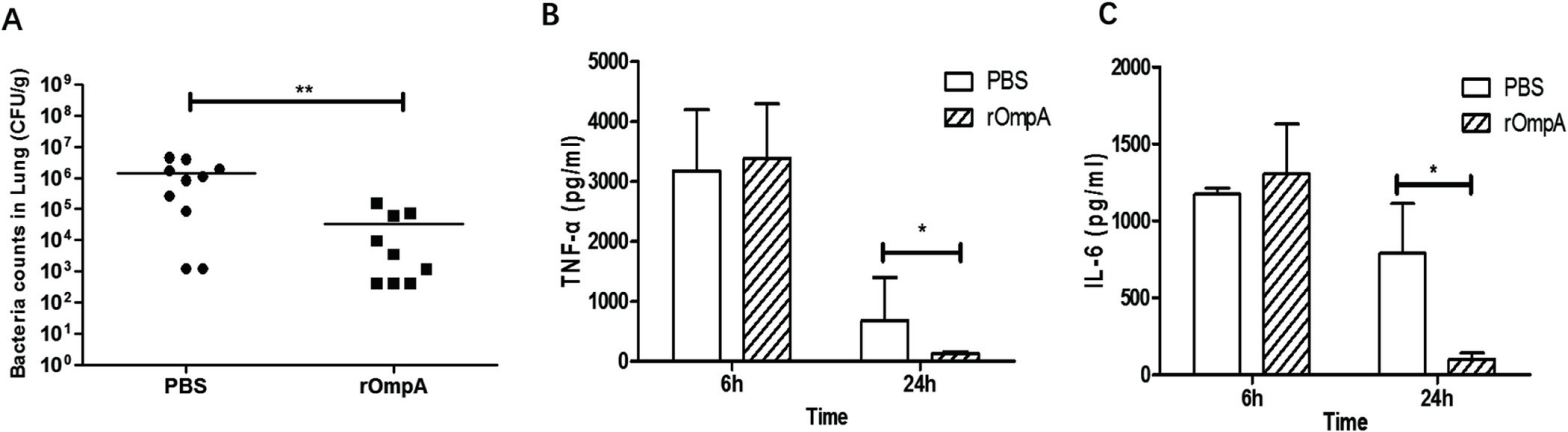
Bacterial loads in lung and pro-inflammatory cytokine levels after intranasal challenge with *S*. *maltophilia*. **A**. Mice were challenged intranasally with *S*. *maltophilia* 10 days after the last immunization. Twenty four hours after the challenge, *S*. *maltophilia* loads in lung homogenate were detected. Data are shown as the median with interquartile range. Results are representative for at least three independent experiments. ** *P* < 0.01 for the rOmpA group (n = 9) compared with control group (n = 10) was analyzed by Mann-Whitney rank test. The levels of TNF-α (**B**) and IL-6 (**C**) in BALF were measured from rOmpA group and control group at 6h and 24h post-challenge. Data are shown as the mean ± SD. Results are representative for three independent experiments. * *P* <0.05 for the rOmpA group compared with control group (PBS) was analyzed by Mann-Whitney rank test (two-tailed).

In order to determine whether intranasal immunization with rOmpA-induced mucosal and systemic immune response can provide protection against respiratory *S*. *maltophilia* infection, we challenged mice with intranasal administration of *S*. *maltophilia* 10 days after the last immunization. Twenty-four hours after the challenge, the *S*. *maltophilia* loads in lung from the rOmpA group were significantly lower than those from the control group (*P*<0.01; **Fig 5A**). Six hours after the challenge, the levels of pro-inflammatory cytokines TNF-α (**Fig 5B**) and IL-6 (**Fig 5C**) in BALF were similar in both groups and reduced 24 hours after the challenge. Notably, TNF-α (**Fig 5B**), and IL-6 (**Fig 5C**) levels in BALF were significantly lower in mice immunized with rOmpA than in the control mice (All *P*<0.05) 24 hours after the challenge.

## Discussion

To the best of our knowledge, the current study first investigated rOmpA-mediated immune response and protection against respiratory *S*. *maltophilia* infection in mice. We found that intranasal immunization with *S*. *maltophilia* rOmpA without additional adjuvant significantly induced systemic and mucosal antibody responses in C57BL/6 mice. The rOmpA immunization-mediated stimulation in serum IgG and BALF mucosal sIgA remained at high levels for a long time. These results indicate intranasal rOmpA immunization may induce strong and sustained systemic and mucosal immune responses in mice. These findings are consistent with the results of previous studies showing that mucosal immunization with recombinant outer membrane protein can induce protective systemic and mucosal immune responses against infections by other Gram-negative bacteria [23, 28, 34, 35]. Serum IgG antibodies are important in systemic humoral immune function, and mucosal sIgA antibodies are the first line of defense against pathogen infection [28]. In this study, mice with intranasal rOmpA immunization also showed significantly increased sIgA levels in the oral, intestinal, and urethral washes, indicating that intranasal delivery of rOmpA may induce immune responses at various mucosal sites. These results are also consistent with the previous study [33].

Previous studies have shown that nasal delivery of an antigen can induce both systemic humoral and cellular immune responses in hosts [33, 36]. In this study, we evaluated the cellular immune response in mice with intranasal rOmpA immunization by analyzing rOmpA-induced IFN-γ, IL-2, IL-4, and IL-17A secretion from primary splenocytes. We found that rOmpA treatment induced significantly and substantially higher IFN-γ secretion from the primary splenocytes of rOmpA-immuned mice than from the primary splenocytes isolated from PBS-immunized mice. However, IL-4 levels were too low to be detected in our study. One of the major cytokines associated with Th1 cells is IFN-γ, while Th2 cells mainly secrete IL-4 [36, 37]. Thus, our results suggest that intranasal rOmpA immunization may mainly induce Th1-type cellular immune response, which activates the phagocytic function of macrophages and natural killer cells and directly stimulates CD8^+^ T cell cytotoxicity. Additionally, Th1 cells can secrete IL-2 as well, which can then further stimulate IFN-γ production and enhance cytotoxicity [33]. In our study, rOmpA-induced increase in IL-2 levels occurred at 12 hours after splenocytes were exposed to rOmpA, whereas rOmpA-induced IFN-γ elevation became significant 24 hours after the exposure. Furthermore, our results showed the IL-17A secretion from primary splenocytes of rOmpA-immuned mice was significantly stimulated by rOmpA. Th17 cells are key mediators of barrier immunity [38], and IL-17 response from mucosal γδ T cells is essential to local immunity [39]. Other studies also reported the IL-17A-mediated production of sIgA in the small intestine and lung [36].

Our findings showed that intranasal immunization with rOmpA induced mucosal and systemic antibody responses and robust Th1- and Th17-mediated cellular immune responses. These immune responses could provide protection against respiratory *S*. *maltophilia* challenge. In fact, our results showed that intranasal immunization with rOmpA did reduce the bacterial burden in mouse lung at 24h after respiratory *S*. *maltophilia* infection challenge in mice. Moreover, respiratory infections could induce cytokine storm, which may lead to severe damage and potentially fatal to the host. In our study, vaccination with rOmpA significantly reduced the levels of TNF-α and IL-6 in BALF at 24 hours after respiratory *S*. *maltophilia* challenge in mice. Therefore, intranasal immunization with rOmpA could protect host against *S*. *maltophilia* infection by reducing bacterial load and preventing excessive pro-inflammatory response in hosts.

## Conclusions

Intranasal immunization with rOmpA of *S*. *maltophilia* without additional adjuvant induced mucosal, systemic, and Th1- and Th17-mediated cellular immune responses, which in turn protected mice against nasal *S*. *maltophilia* infection. Therefore, rOmpA could be a promising and effective vaccine candidate against *S*. *maltophilia* infection. In fact, intranasal vaccination may offer an alternative approach to current strategies since it induces a mucosal as well as a systemic immune response.

### Ethics approval and consent to participate

The study was performed in accordance with national guidelines and regulations, and all animal experiments were approved by Institutional Animal Care and Use Committee of Academy of Military Medical Sciences (AMMS).
